# Identification of N-Glycosylation in Hepatocellular Carcinoma Patients’ Serum with a Comparative Proteomic Approach

**DOI:** 10.1371/journal.pone.0077161

**Published:** 2013-10-15

**Authors:** Yingnan Huang, Hao Wu, Ruyi Xue, Taotao Liu, Ling Dong, Jun Yao, Yang Zhang, Xizhong Shen

**Affiliations:** 1 Department of Gastroenterology, Zhongshan Hospital of Fudan University, Shanghai, China; 2 Institutes of Biomedical Sciences, Fudan University, Shanghai, China; 3 Department of Chemistry, Fudan University, Shanghai, China; West German Cancer Center, Germany

## Abstract

**Aim:**

This study is to explore the different expressions of serum N-glycoproteins and glycosylation sites between hepatocellular carcinoma (HCC) patients and healthy controls.

**Method:**

We combined high abundant proteins depletion and hydrophilic affinity method to enrich the glycoproteins. Through liquid chromatography-tandem mass spectrometry (LC-MS/MS), we extensively surveyed different expressions of glycosylation sites and glycoproteins between the two groups.

**Result:**

This approach identified 152 glycosylation sites and 54 glycoproteins expressed differently between HCC patients and healthy controls. With the absolute values of Pearson coefficients of at least 0.8, eight proteins were identified significantly up or down regulated in HCC serum. Those proteins are supposed to be involved in several biological processes, cellular components and molecular functions of hepatocarcinogenesis. Several of them had been reported abnormally regulated in several kinds of malignant tumors, and may be promising biomarkers of HCC.

**Conclusion:**

Our work provides a systematic and quantitative method of glycoproteomics and demonstrates some key changes in clinical HCC serum. These proteomic signatures may help to unveil the underlying mechanisms of hepatocarcinogenesis and may be useful for the exploration of candidate biomarkers.

## Introduction

Hepatocellular carcinoma (HCC) is the ﬁfth most common cancer and the third leading cause of cancer death worldwide[1]. A 10-year survey (1990-2001) conducted in China indicates that HCC ranks ﬁrst among chronic diseases for the social cost and burden in the World Health Organization (WHO) “disability-adjusted life year” list[2]. The 5-year survival rate of all HCC is less than 5%, placing it among the cancers with worst prognosis[3]. Its high mortality is mainly attributed to the difficulty of early diagnosis.

Alpha-fetoprotein (AFP) is widely used for HCC’s surveillance and detection test among patients with cirrhosis. Other serologic biomarkers such as lectin-bound AFP (AFP-L3), des-γ carboxyprothrombin (DCP) and Golgi protein 73 (GP73) are also widely used in clinical practice to detect HCC[4-7]. However, their sensitivities and specificities are not adequate. Meanwhile, AFP-negative HCC is frequently observed. Thus, development of novel biomarkers for early detection remains an important target before a breakthrough appears on HCC surveillance.

Glycosylation is one of the most prominent posttranslational protein modiﬁcations and plays a major role in the assembly of complex multicellular organs and organisms. This modiﬁcation is involved in many cellular functions including cell-cell and receptor-ligand interactions, immune response, apoptosis, and pathogenesis of many diseases. Cancer cells are known to express aberrant glycosylation patterns such as branching of N-glycans changes, expression and glycosylation of mucins changes, sialic acid expression changes, Lewis structures overexpression, etc. [8,9]. Many cancer biomarkers frequently used clinically are glycoproteins, such as AFP, prostate-specific antigen (PSA) and carcinoembryonic antigen (CEA). Cancer glycoproteomics has been a new direction for cancer diagnosis and biomarker detection. Typically, carbohydrates are linked to serine or threonine residues (O-linked glycosylation) or to asparagine residues (N-linked glycosylation). N-linked glycosylation sites generally fall into the N-X-Ser/Thr (N-X-S/T) sequons, in which X denotes any amino acid except proline. N-glycosylation is widespread in extracellular places[10]. Glycosylated proteins, N-linked glycosylation in particular, are prevalent in proteins destined for extracellular environments[11].

With the coupling of advanced capillary-based LC-separations online with MS analyses, proteomics practice has become much easier than before. Label free relative quantitation, which does not require up-front isotopic labeling and permits retrospective comparison, is gaining interest. With these methods, we applied a comparative glycoproteomics analysis to the serum of HCC patients and healthy controls in this study.

## Materials and Methods

### 1: Chemicals and Materials

Bradford assay reagent, sodium Proteo-Miner™ Protein Enrichment Kits were obtained from Bio-Rad. 3000 Da MWCO spin columns were from Millipore. Sepharose CL-4B was from Amersham Bioscience. Sequencing grade modiﬁed trypsin was from Promega. PNGase F was from New England Biolabs. C18 spin columns were from Waters. The protein assay kits were from Shanghai Sangon. All other chemicals were purchased from Shanghai Sangon.

### 2: Ethics Statement

In our experiment, we collected peripheral blood samples from newly diagnosed HCC patients and healthy controls, 4 ml each. All the participants provided their written informed consents to participate in this study. The samples’ collection, experimental scheme and informed consents had all been approved by the ethics committee of biomedical research, Zhongshan Hospital, Fudan University. The permit number is 2011-29.

### 3: Samples Collection

In Zhongshan hospital, 19 newly diagnosed HCC patients and 19 healthy controls were included during March, April and May of 2012, with 17 male and 2 female (neither of them were in menstrual cycle when their serum were collected) in each group. HCC patients had not had any special treatments, such as operations, interventional therapies or radiotherapies before. HCC patients were diagnosed by one imaging modality (ultrasound [US], magnetic resonance imaging [MRI], or computed tomography [CT]) and pathological evidence. 14 of the 19 patients had positive AFPs with 20 ng/ml as the threshold value. 17 of the 19 patients has positive HBsAg. At the time of diagnosis, 18 of the 19 patients were also found to combine liver cirrhosis. Healthy volunteers were from outpatient department with no reported gastrointestinal and hepatobiliary diseases. None of the 38 persons had hepatitis C virus (HCV) infections. The tumor staging is according to the 6th edition of the American Joint Committee on Cancer (AJCC)/ International Union Against Cancer (UICC) tumour-node-metastasis (TNM) system (TNM-6)[12]. The clinical and demographic characteristics of HCC cases and clinical controls are summarized in Table 1. The signiﬁcances for the differences in age and gender between cases and controls were determined using Fisher’s exact test. Neither of the demographic characteristics was signiﬁcantly different between the two groups.

**Table 1:** The clinical and demographic characteristics of HCC cases and clinical controls.

	HCC patients			Healthy Controls	
	N	%		N	%
Age					
50-54	2	10.53		3	15.79
55-59	5	26.32		5	26.32
60-64	4	21.05		4	21.05
65-69	5	26.32		3	15.79
70-74	3	15.79		3	15.79
75-79	0	0		1	5.26
Gender					
Male	17	89.47		17	89.47
Female	2	10.53		2	10.53
Tumor Stage					
I	10	52.63		0	0
II	0	0		0	0
III	8	42.05		0	0
ⅢA	1	5.26		0	0
ⅢB	1	5.26		0	0
ⅢC	6	31.58		0	0
IV	1	5.26		0	0
Clinical settings combined					
HBsAg (+)	17	89.47		0	0
Anti-HCV (+)	0	0		0	0
Liver cirrhosis	18	94.74		0	0

4 ml of peripheral blood was obtained from each participant. After collection, they were left stood for 2 h at room temperature and then centrifuged at 3000 g and 4 °C for 5 min. Serum samples were stored at -80 °C until analysis.

### 4: Samples Pretreatment

#### (1): Removals of the High Abundant Proteins

We used Proteo-Miner™ Protein Enrichment Kits to remove high abundant serum proteins. 1 ml serum for each sample was freezing-thawed. Proteo-Miner™ Protein Enrichment Kits were used to remove their high abundant serum proteins. The lower abundant serum proteins were contained in the eluate fractions. They were next desalted and concentrated through buffer exchange with 50 mM NH_4_HCO_3_ using 3000 Da MWCO spin columns. Total protein concentrations were determined. 

#### (2): Glycoproteins Enrichment

In this step, we used the hydrophilic affinity method to separate the glycoproteins fromother proteins for enrichment. The method has been previously described[13-15]. For each sample, about 100 µg of the protein solution was added to a microcentrifuge tube containing 15 µL of sepharose. Then, 1 mL of organic solvent mixture containing butanol/ethanol/water (4:1:1 by volume) was added to the protein/sepharose mixture. The resulting mixture was gently mixed for 1 h and then centrifuged for 5 min at 4 °C. The supernatant was removed. The pellet was washed three times with the same organic solvent. Glycoproteins were extracted by incubating the pellet in 50% ethanol aqueous solution for 30 min. The samples were vortexed and then centrifuged. For each sample, supernatant was transferred to another new microcentrifuge tube. The same extraction procedure was repeated once. Finally, the combined supernatant was dried in a vacuum centrifuge and then resolved in deionized-water.

#### (3): In-solution Digestion

The concentrated proteins were heated at 100 °C for 10 min. After the samples had cooled down to room temperature, dithiothreitol (DTT) at a ﬁnal concentration of 10 mM was introduced into the solution. The samples were incubated at 57 °C for 30 min. To prevent disulﬁde bond formation, cysteine residues were alkylated by iodoacetamide (IAA). IAA was added to the sample solution at 20 mM ﬁnal concentration and incubated 30 minutes in the dark at room temperature for fully alkylation. Then the sample solution was diluted 10-fold with 50 mM NH_4_HCO_3_ buffer and mixed with trypsin at 50:1. The mixture was then incubated overnight at 37 °C. Glycopeptides were then deglycosylated with PNGase F (500 units/µL) at a concentration of 1 µL of PNGase F per 1 mg of crude proteins in 100 mM NH_4_HCO_3_ (pH 8.0).

#### (4): Removal of Salt

All digested peptide mixtures were passed over C18 columns to remove extra DTT and salt. Peptides were eluted from the column with 80% acetonitrile (ACN) in 0.1% triﬂuoroacetic acid (TFA) and then dried in a vacuum centrifuge for later use.

**Figure 1:**
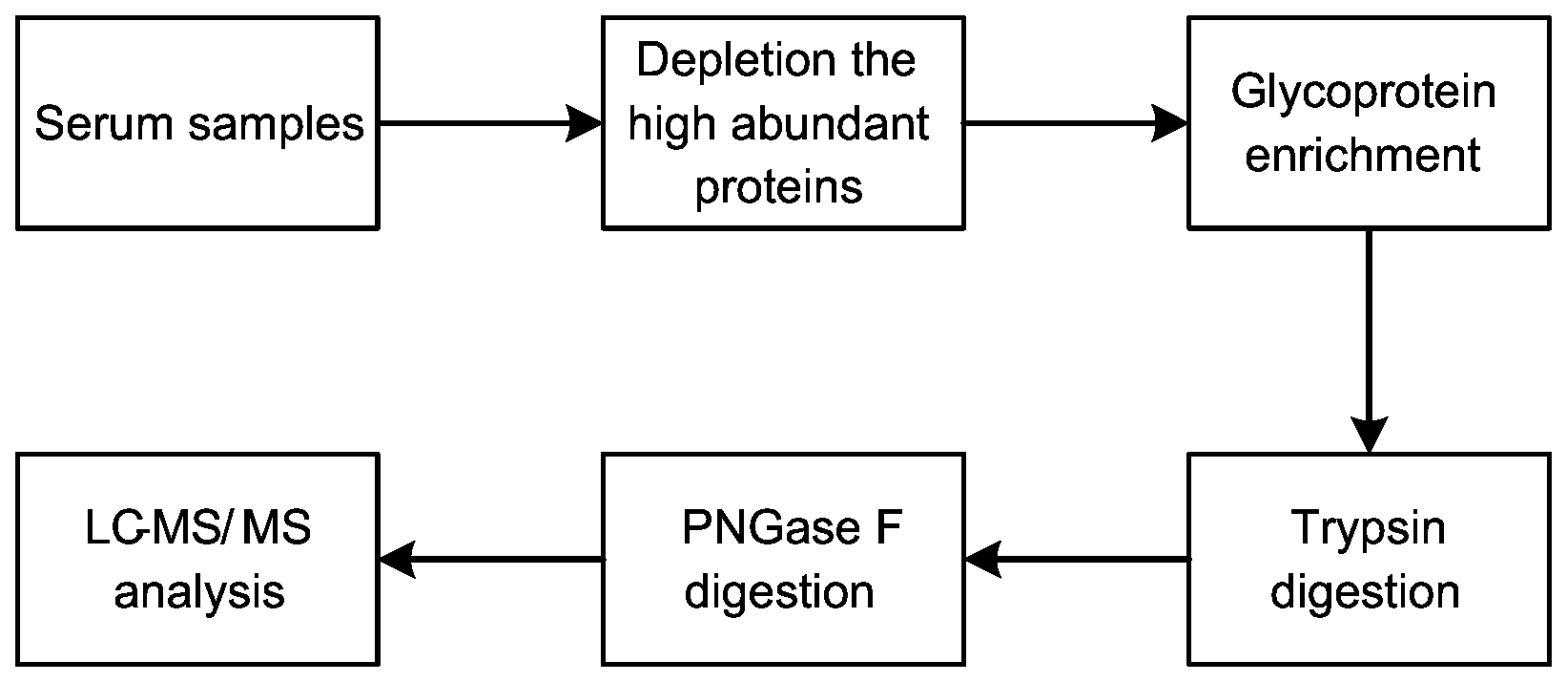
Schematic illustration of serum samples processing.

The process above was briefly shown in Figure 1.

### 5: LC-MS/MS Analysis

For each individual sample, nano-LC MS/MS experiment was performed on an HPLC system composed by 2 LC-20AD nano-flow LC pumps and 1 LC-20AB micro-flow LC pump (all from Shimadzu Corporation, Tokyo, Japan) connected to an LTQ-Orbitrap mass spectrometer (Thermo Electron Corporation, San Jose, CA). Sample injection was done via an SIL-20 AC auto-sampler (Shimadzu Corporation, Tokyo, Japan) and loaded onto a CAPTRAP column (0.5 x 2 mm, MICHROM Bioresources Inc., Auburn, CA) for 5 min at a flow rate of 60 μL/min. The sample was subsequently separated by a PICOFRIT C18 reverse-phase column (0.075 x 100 mm, New Objective Inc., Woburn, MA) at a flow rate of 500 nL/min. The mobile phases consisted of 5% acetonitrile with 0.1% formic acid (phase A and the loading phase) and 95% acetonitrile with 0.1% formic acid (phase B). To achieve proper separation, a 90-min linear gradient from 5 to 45% phase B was employed. The separated sample was introduced into the mass spectrometer via a 15 μm silica tip (New Objective Inc., Woburn, MA) adapted to a DYNAMIC nano-electrospray source (Thermo Electron Corporation, San Jose, CA). The spray voltage was set at 1.9 kV and the heated capillary at 220°C. The mass spectrometer was operated in data-dependent mode and each cycle of duty consisted one full- MS survey scan at the mass range 400~2000 Da with resolution power of 100,000 using the Orbitrap section, followed by MS2 experiments for 8 strongest peaks using the LTQ section. The AGC expectation during full-MS and MS/MS were 500000 and 10000, respectively. Peptides were fragmented in the LTQ section using collision-induced dissociation with helium and the normalized collision energy value set at 35% and previously fragmented peptides were excluded for 30 s.

### 6: Bioinformatics Analysis

**1 Database search** Protein searches were performed with MaxQuant 1.3.0.5 against the IPI databases for human version 3.68 (downloaded from ftp://www.ebi.ac.uk) with the following criteria: 2 possible missed cleavage sites, peptide mass tolerance of 20 ppm, fragment mass tolerance of 0.50 Da, oxidized Met and acetylated N-term were considered as possible modifications. The acceptance criteria for peptide identifications was the rate of false positive identification should be less than 1%, thus increasing the confidence of the identified proteins.
**2 Technological reliability verification** The N-glycosylation sequences were submitted to the NetNGlyc 1.0 Server (http://www.cbs.dtu.dk/services/NetNGlyc/), CBS information tools. N-Glycosylation sites in human proteins were predicted using artificial neural networks that examined the sequence context of N-X-S/T sequons.
**3 Quantitative analysis** After submitting all the peptides into the Maxquant database, the correlated leading proteins were identified. For one protein, the peak area stands for its quantity. According to this principle, a pairwise comparison for every protein between the two groups was carried out. Pearson coefficient was used to evaluate the significance of differential expression. In either group, an absolute value of the coefficient ≥0.8 was considered as the criteria of significantly differential expression between the two groups.
**4 Gene ontology (GO) analysis** All the identified proteins were submitted to the DAVID database. Using the function annotation tool, GO analysis, including biological process (BP), cellular component (CC) and molecular function (MF) analysis, was performed. The glycoproteins were sorted according to different GO items and compared with the GO annotation of the whole putative N-glycoproteome from human. Corresponding enrichment tests were carried out.

## Results

### 1: Samples Pretreatment

**Figure 2:**
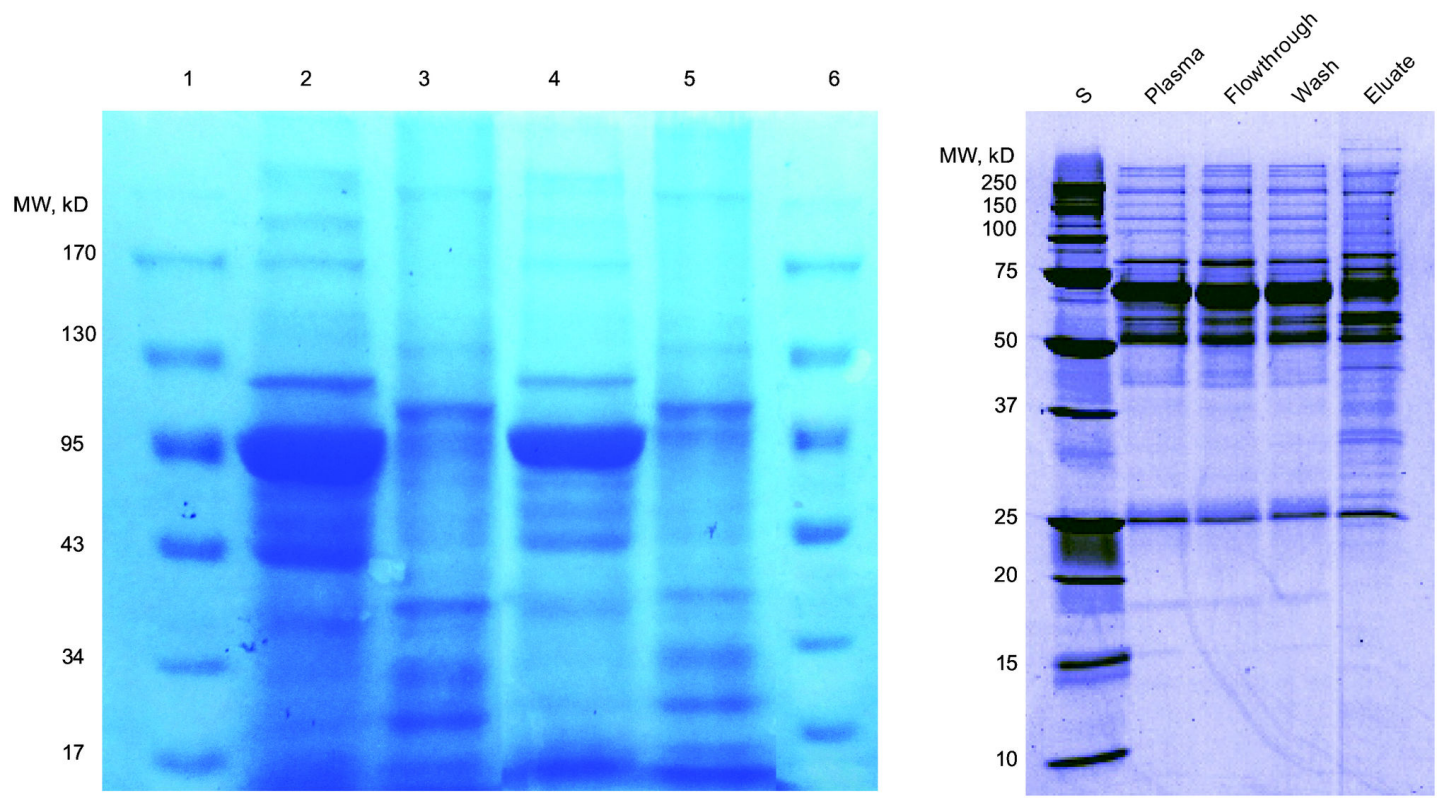
Figures of SDS-PAGE gels stained with Coomassie blue, both before and after depletion of the high abundant protein. The left one is the result of our samples Lane 2, 3 and 4 are from a HCC sample; lane 5, 6 and 7 are from a healthy control. Lane 1 and 8 are figures of the marker used in our experiment; lane 2 and 5 are figures of serum; lane 3 and 6 are figures of eluate fraction, out of which high abundant proteins had been depleted. The right one is the kit-treated plasma provided by the website of the Proteo-Miner™ Kits (http://www.bio-rad.com/webroot/web/pdf/lsr/literature/Bulletin_5632.pdf). The 1st lane (S) is the figure of the marker; the 2nd lane is the figure of plasma; the 5th lane is the figure of eluate fraction, out of which high abundant proteins had been depleted.

High abundant proteins in serum were depleted using Proteo-Miner™ Protein Enrichment Kits. SDS-PAGEs were applied to the samples before and after depletion to observe the kits’ efficiency. Among them, two samples’ results were shown in Figure 2. Near the 72 kDa area, the most abundant band, which is most likely albumin, was markedly reduced in the eluted fraction. In addition, a series of bands appeared in the range below 50 kDa, which represent proteins that have been concentrated. The results were similar as the result provided by the website of the Kits.

### 2: LC-MS/MS and Bioinformatics Analysis

#### (1): Database search results

**Figure 3:**
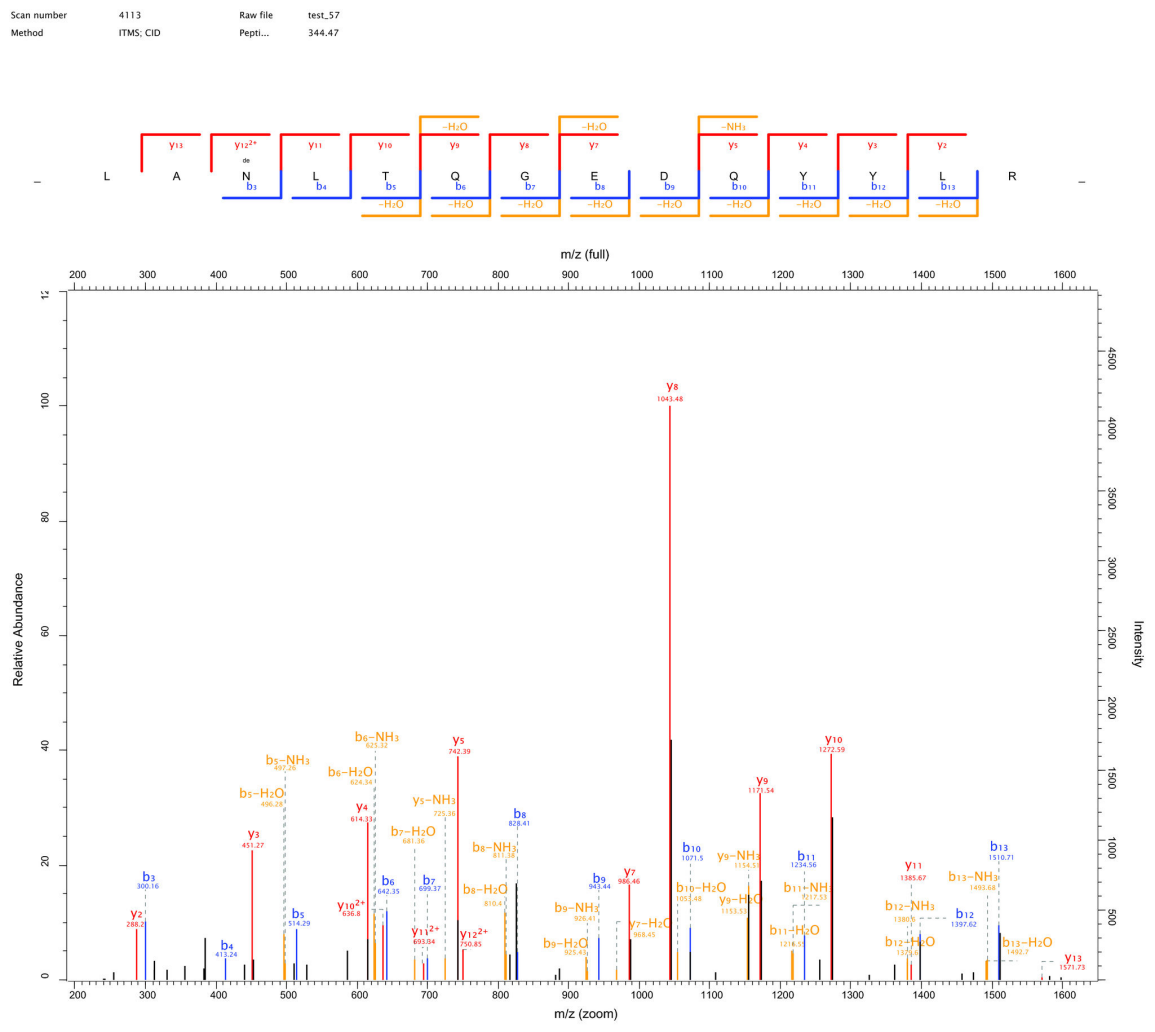
Nano-LC-MS/MS mass spectrum of N-glycosylated peptide LANdeLTQGEDQYYL from CLU. The de denotes the residue site of N-glycosylation.

The collected peptides were analyzed by Nano-LC MS/MS (HPLC-LTQ-Orbitrap). By searching in the database of MaxQuant 1.3.0.5, a total of 152 glycopeptides were identiﬁed within 58 leading proteins. Figure 3 shows a representative nano-LC MS/MS spectrum from clusterin (CLU, also named Apo-lipoprotein J). The glycopeptides account for 10% of the total peptides identified. Among the 152 identified glycosylation sequons, there were 88 N-X-S/T sequons and 64 non-N-X-S/T sequons.

#### (2): Technological reliability verification

We submitted the N-glycosylation sequences to the CBS information tools. 69 of the 88 N-X-S/T sequons and 53 of the 64 non-N-X-S/T sequons were positively predicted as N-Glycosylation sites by the database. Those left were negatively predicted, most of which were with less confirmation (-, 16/19 in N-X-S/T sequons vs. 8/11 in Non- N-X-S/T sequons. The others were - - or - - -, which meant higher certainty). The positive prediction ratios were similar in the N-X-S/T sequons and non-N-X-S/T sequons, which suggested the consensus between the two groups and the reliability of our methodology (Table 2).

**Table 2:** Prediction of the N-Glycosylation sites using the Net-N-Glyc Server, the CBS information tools.

Test result / Prediction	Glycosylation sequons	
	N-X-S/T	Non- N-X-S/T
-	16	8
--	1	1
---	2	2
Subtotal	19 (21.59%)	11 (17.19%)
+	35	24
++	26	21
+++	8	8
Subtotal	69 (78.41%)	53 (82.81%)
Total	88	64

#### (3): Quantitative analysis

To identify glycopeptides with differential expression between HCC patients and healthy controls, we performed a pairwise comparison using Pearson coefficient. An absolute value of the coefficient >=0.8 suggested a linear correlation between a peptide expression and the health condition, and further, differentially regulated glycosylation level in either group (the closer to -1, the higher expression in HCC patients; the closer to 1, the higher expression in healthy controls). A total of 11 glycosylation peptides met the condition and were listed below (Table 3).

**Table 3:** 11 glycosylation peptides and their corresponding leading proteins differentially expressed between HCC patients and healthy controls (Pearson coefficient<0, up- regulated in HCC patients; 0, down-regulated in HCC patients).

**Protein**	**Position**	**Pearson Coefficient**	**P value**	**Description**
IPI00022229	185	-1	N/A	APOLIPOPROTEIN B-100
IPI00553177	70	0.855646744	0.1443	ISOFORM 1 OF ALPHA-1-ANTITRYPSIN.
IPI00235412	596	0.89747284	0.2908	64 KDA PROTEIN
IPI00235412	615	0.99820244	0.0381	64 KDA PROTEIN
IPI00434968	3	0.99820244	0.0381	SMALL UBIQUITIN-RELATED MODIFIER 4
IPI00896419	573	1	N/A	ISOFORM 1 OF INTER-ALPHA-TRYPSIN INHIBITOR HEAVY CHAIN H4.
IPI00896419	577	1	N/A	ISOFORM 1 OF INTER-ALPHA-TRYPSIN INHIBITOR HEAVY CHAIN H4.
IPI00644977	557	1	N/A	COMPLEMENT FACTOR H-RELATED PROTEIN 4 ISOFORM 2 PRECURSOR
IPI00029739	1029	1	N/A	ISOFORM 1 OF COMPLEMENT FACTOR H
IPI00400826	217	1	N/A	ISOFORM 2 OF CLUSTERIN.

Among them, negative coefficients were found in one peptides within one leading protein, apolipoprotein A-IV (ApoA-IV), and suggested an up-regulated glycosylation level in HCC patients. Positive coefficients were found in the other 9 glycosylation peptides within 7 leading proteins, including isoform 1 of α-1 antitrypsin (A1AT), 64 kDa protein, small ubiquitin-related modifier (SUMO), isoform 1 of inter-α-trypsin inhibitor heavy chain H4 (ITIH4), complement factor H-related protein 4 isoform 2 (CFHR-4), isoform 1 of complement factor H (CFH-1), isoform 2 of CLU, and suggested a down-regulated glycosylation level in HCC patients compared to the healthy controls. 

#### (4): GO Analysis

**Figure 4:**
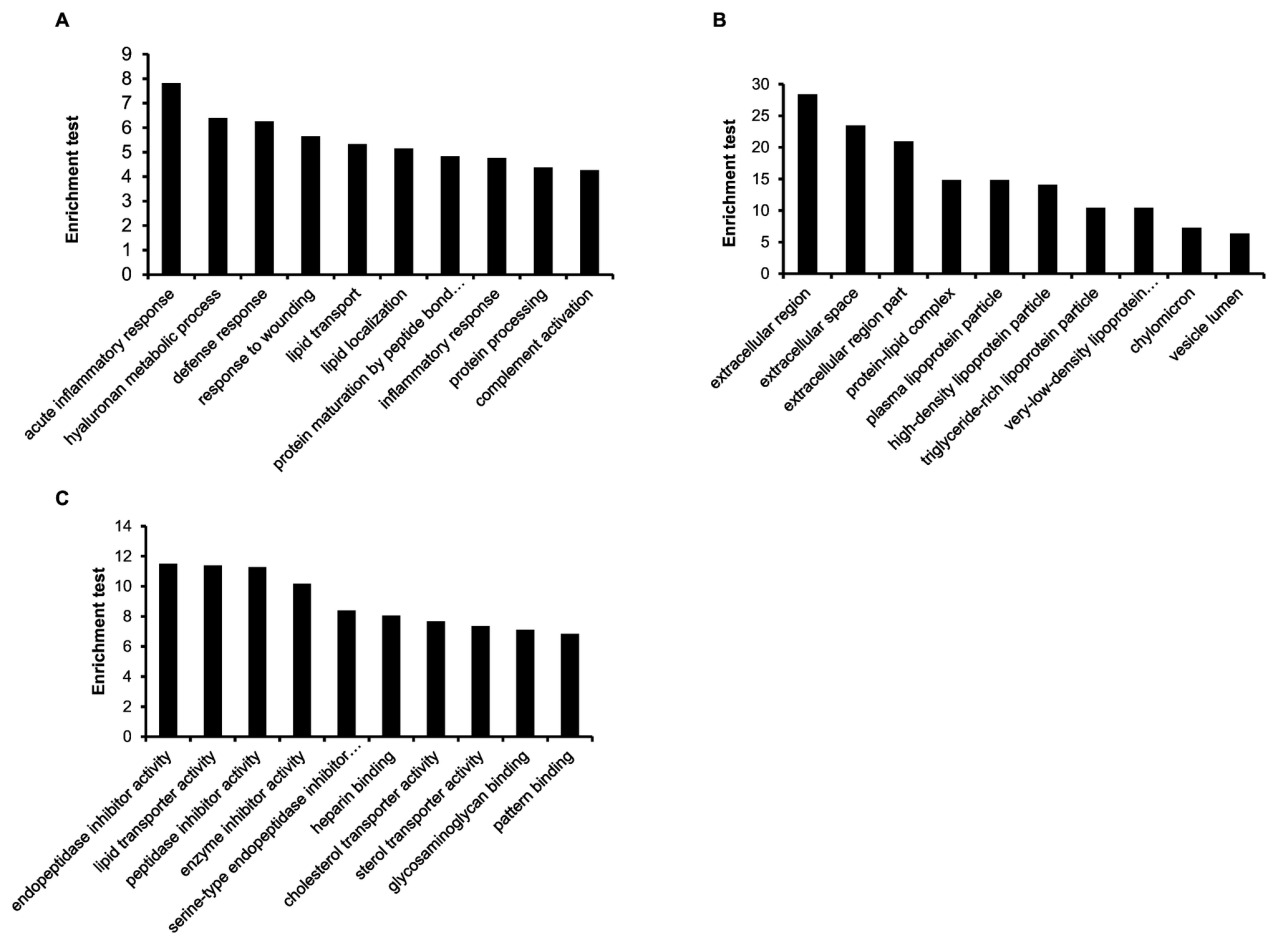
GO annotation enrichment tests. **A**. GO annotation of Biological process (BP). **B**. GO annotation of Cellular component (CC). **C**. GO annotation of Molecular function (MF).

All the identified proteins were submitted to the DAVID database. GO analysis and corresponding enrichment tests were carried out (Figure 4). The BP analysis indicated that these proteins are mainly involved in acute inflammatory response, hyaluronan metabolic process; The CC analysis indicated that these proteins are mainly involved in extracellular region, extracellular space and extracellular region part; The MF analysis indicated that these proteins are mainly involved in endopeptidase inhibitor activity, lipid transporter activity, and peptidase inhibitor activity. They all accorded with glycoproteins’ functions and cellular localization as secreted proteins.

## Discussion

HCC is the fifth leading cause of cancer death worldwide and is one of the most common malignancies in China. The prognosis of HCC patients remains poor, especially for those at advanced stages of the disease. Indeed, identiﬁcation of early-stage HCC may be the most promising approach for reducing its mortality. Here we analyzed HCC patients’ serum using proteomic approaches and made an effort to search promising biomarkers.

Peripheral blood carries various proteins, many of which are secreted by cells during different physiology or pathology processes. Therefore, serum proteins can reflect the general situation of the human organism. However, high abundant proteins such as albumin and IgG constitute approximately 60–80% of the total serum proteins[16,17], making the detection of medium- and low abundant proteins extremely challenging. In our study, we applied the Proteo-Miner™ Protein Enrichment Kits and hydrophilic afﬁnity (HA) method for enrichment of proteins.

The Proteo-Miner™ (also named Equalizer beads) technology uses a combinatorial library of hexapeptides bound to beads. Due to the tremendous ligand diversity within the library, there is theoretically one ligand for every protein, antibody, and peptide present in the starting material. The equalizer beads, as their name implies, work by binding proteins until saturation is reached. The high-abundant proteins will reach saturation quickly, while the lower-abundant proteins continue to bind. The excess unbound proteins will be washed away in the procedure. This results in a dramatic depletion of the most abundant proteins, with a concurrent concentration of the middle- to low-abundant proteins. It can address the problem of wide dynamic range of the proteins in serum and, more importantly, overcome the loss of proteins by the removal of “total” albumin via immunodepletion methods, for albumin are frequently functioning as carriers and transporters of important proteins [16,18,19].

The mechanism of HA is to immobilize glycoproteins on solid phase, based on the hydrogen bonding between carbohydrate oxygens of the gel-matrix (sepharose or cellulose) and glycoproteins. Apart from HA, hydrazide chemistry (HC) and Boronate affinity can also be used for enrichment. Of course, due to the complexity and heterogeneity of glycosylation, none of these methods can capture all the glycoproteins in complex biological samples. Fewer steps and no preferences to particular glycoforms make HA frequently used in glycoproteins enrichment[13,14]. Glycopeptides are usually present in relatively low abundance (2-5%) in the enzymatic peptides of full glycoprotein[20]. In our experiment, the glycopeptides account for 10% of the total peptides identified, indicating the efficiency of our enrichment.

PNGase F cleaves nearly all types of N-glycans from the polypeptide backbones and also possesses an additional amidase activity during this process. Therefore, PNGaseF converts asparagine to aspartic acid during the cleavage reaction. This results in a 1 Da mass shift detectable by most of the currently available mass spectrometers, especially by advanced instrument with distinguishable and superior mass accuracies.

In our study, proteins found with lower abundant levels in HCC serum compared to normal serum included CLU, A1AT, ITIH4, FHR-1 and SUMO. Apo B-100 was found with higher abundant levels in HCC sera compared to normal sera. Their abnormal expression had been found associated with HCC or other cancers in previous studies, although the polarity of the changes between the cases and the controls may differ. 

**CLU** CLU is a secretory glycoprotein expressed in several tissues and present in all human ﬂuids. It has been involved in a wide range of physiological and pathophysiological processes important for carcinogenesis and tumor growth, including lipid transportation and redistribution, apoptosis, cell cycle regulation, DNA repair, folding of damaged extracellular proteins, cell adhesion and aggregation, membrane recycling, complement regulation, tissue remodeling and immune system regulation [21]. The expression status of CLU might change in many human cancers, such as gastric cancer[22], colon cancer[23], pancreatic cancer, prostate cancer[24], breast cancer[25], lung cancer and renal cell carcinoma[26]. However, a lot of data reported in the literature appear contradictory with each other even in the same kind of cancer.

In our study, glycosylated CLU was found down-regulated in the serum of HCC patients compared to the healthy controls, which is consistent with some previous studies, and meanwhile, in contrast with some other studies. Take an overall consideration, the role of CLU may be more complex than simply one-way. 

CLU has been described as dual functional---both pro-apoptotic and anti-apoptotic. It was discovered to include different forms—secreted CLU and intracellular CLU (nuclear CLU and cytoplasmic CLU)[27]. The secreted form of CLU has been shown to be cytoprotective and may confer increased resistance to killing by anti-cancer drugs or enhance tumor cell survival in speciﬁc niches, whereas the nuclear form is pro-apoptotic[23,27]. These discrepancies may be related to the fact that CLU has various glycosylation forms. Some researchers have concluded that different isoforms of CLU could participate in processes that may have opposite effects, such as apoptotic and anti-apoptotic roles. In our study, CLU with a certain glycosylate sites was found to be down-regulated in the patients’ serum. We may deduce that the protein with a certain glycosylation form, rather than the total secreted protein, varied.

One relevant study was published by Pucci et al.[28] suggesting that the shifting balance between CLU forms (nuclear CLU /secreted CLU) during tumor progression, by affecting the fate of the cell, seems to be strongly influenced by the metabolic shift occurring in the different steps of tumor progression. Meanwhile, the special isoforms may be promising biomarkers for cancers. Similar situation was identified in the study by Rodríguez-Piñeiro, et al.[29], CLU in the serum of colorectal cancer patients was analyzed using proteomic techniques and was identified to have several different isoforms, each with a different glycosylation level. Two-dimensional electrophoretic analysis verified that point.

Other proteins found to be of lower abundant in HCC patients included A1AT, ITIH4, FHR-1 and SUMO. 

**ITIH4** ITIH4 is a 120 kDa glycoprotein. The family of inter-alpha-trypsin inhibitors (ITI) is composed of serine protease inhibitors that are assembled from two precursor proteins: a light chain and either one or two heavy chains[30]. ITIH4 is a member of the family of heavy chains (ITIHs). There is strong evidence that all members of the ITI family play important roles in malignant processes[31-33]. A systematic expression analysis using Cancer Profiling Array – CPA (Product No. 631761; Clontech, Heidelberg, Germany, contains spotted tumor cDNAs and corresponding normal tissue from the same patient) showed that down-regulation of ITIH4 in cancer tissue was detected in tumors of the kidney (95%), stomach (63%) ovary (57%), colon cancer (54%), lung cancer (52%), rectum cancer (50%), and prostate cancer (75%), suggesting that the down-regulation of IHIT4 may be associated with initiation and/or progression of these malignancies[34].

**CFHR-4** CFHR-4 is a kind of secreted plasma glycoprotein produced primarily in the liver. It is one of the six members of the factor H protein family[35]. Qualitative urine test of the factor H is one way to detect bladder tumor[36]. In our study, we found the CFHR-4 to be decreased in HCC serum. It was similar to one previous study by Fu et al. through a comparative proteomic approach between metastatic and non-metastatic HCC[37]. They also found this protein down-regulated in the serum of metastatic HCC patients through MALDI-TOF/TOF analysis.

Proteomics technology and a combination of serological methods in serum screening have provided a technological advancement for identifying tumor markers. These methods are used frequently in a variety of current studies. With the continual development of proteomics approaches, the establishment of accurate panels of biomarkers for specific diseases is achievable. Meanwhile, by discovering more and more potential biomarkers and developing our understanding on their roles in the pathophysiology of HCC, such strategies have enormous potential in influencing the development of new treatments and more precise screening for this disease.

In our study, we paid attention to the homogeneity between the two groups, such as age and gender. However, there are still limitations. Further verification may be needed especially for the promising markers before deeply exploration into their related mechanism. Improvements in reproducibility of serum proteomics are still needed.

## Conclusion

Our work provided a systematic and quantitative analysis and demonstrated key changes in clinical HCC serum. These proteomic signatures could help to unveil the underlying mechanisms of hepatocarcinogenesis and may be useful for the discovery of candidate biomarkers.
